# Supplementary material for: Intakes of Folate, Vitamin B6, and Vitamin B12 in Relation to All-Cause and Cause-Specific Mortality: A National Population-Based Cohort
Source: Nutrients. 2022 May 27;14(11):2253. doi: 10.3390/nu14112253 (PMC9182598; doi:10.3390/nu14112253)
Supplement: Supplementary file 1 [file nutrients-14-02253-s001.zip › nutrients-1675045-supplementary.pdf]

Table S1. Stratified analyses of the associations between dietary folate intake with all-cause and cause-specific mortality in men

	Quartile of dietary folate intake				P_{trend}	$P_{interaction}$
	Q1	Q2	Q3	Q4		
**All-cause Mortality**						
**Age**						0.138
<65y	Ref	1.00(0.90-1.10)	0.90(0.80-1.02)	0.85(0.72-0.99)	0.026	
≥65y	Ref	0.85(0.79-0.93)	0.84(0.77-0.92)	0.72(0.64-0.80)	<0.001	
**Current smoker**						0.252
Yes	Ref	0.96(0.85-1.08)	0.84(0.72-0.97)	0.86(0.72-1.03)	0.010	
No	Ref	0.94(0.88-1.01)	0.88(0.81-0.96)	0.77(0.69-0.86)	<0.001	
**Current drinker**						0.557
Yes	Ref	0.95(0.88-1.02)	0.84(0.77-0.93)	0.79(0.71-0.89)	<0.001	
No	Ref	0.93(0.83-1.03)	0.90(0.80-1.01)	0.82(0.70-0.96)	0.010	
**BMI**						0.077
<30 kg/m²	Ref	0.92(0.86-0.99)	0.85(0.78-0.92)	0.79(0.71-0.88)	<0.001	
≥30 kg/m²	Ref	0.88(0.77-0.99)	0.87(0.75-1.01)	0.79(0.66-0.95)	0.006	
**Cardiovascular Mortality**						
**Age**						0.015
<65y	Ref	0.93(0.74-1.17)	0.75(0.56-1.02)	0.89(0.61-1.29)	0.146	
≥65y	Ref	0.74(0.63-0.87)	0.78(0.65-0.94)	0.49(0.39-0.62)	<0.001	
**Current smoker**						0.389
Yes	Ref	0.80(0.62-1.03)	0.62(0.45-0.85)	0.55(0.36-0.85)	<0.001	
No	Ref	0.82(0.71-0.96)	0.79(0.66-0.94)	0.59(0.47-0.75)	<0.001	
**Current drinker**						0.450
Yes	Ref	0.82(0.69-0.97)	0.64(0.52-0.78)	0.60(0.47-0.77)	<0.001	
No	Ref	0.84(0.66-1.05)	1.10(0.86-1.42)	0.75(0.51-1.11)	0.650	
**BMI**						0.226
<30 kg/m²	Ref	0.81(0.70-0.95)	0.75(0.63-0.90)	0.60(0.47-0.77)	<0.001	
≥30 kg/m²	Ref	0.73(0.56-0.94)	0.66(0.48-0.91)	0.55(0.37-0.83)	0.001	
**Cancer Mortality**						
**Age**						0.580
<65y	Ref	1.00(0.82-1.22)	1.01(0.79-1.28)	0.82(0.59-1.14)	0.334	
≥65y	Ref	0.82(0.69-0.98)	0.79(0.65-0.96)	0.65(0.51-0.82)	<0.001	
**Current smoker**						0.770
Yes	Ref	0.84(0.64-1.10)	0.81(0.58-1.11)	0.74(0.49-1.12)	0.085	
No	Ref	0.96(0.82-1.11)	0.92(0.77-1.09)	0.72(0.58-0.91)	0.011	
**Current drinker**						0.294
Yes	Ref	1.00(0.85-1.17)	0.94(0.78-1.13)	0.73(0.57-0.93)	0.029	
No	Ref	0.74(0.58-0.93)	0.7(0.54-0.91)	0.72(0.51-1.00)	0.008	
**BMI**						0.890
<30 kg/m²	Ref	0.91(0.78-1.05)	0.87(0.73-1.04)	0.63(0.49-0.80)	<0.001	
≥30 kg/m²	Ref	0.76(0.58-1.01)	0.89(0.65-1.21)	0.86(0.59-1.24)	0.340	

Adjusted for age, race/ethnicity, BMI, family income-poverty ratio, smoking status (not for smoke stratified analysis), drinking (not for drinking stratified analysis), leisure-time physical activity, total energy intake, diabetes, hypertension, and cardiovascular disease.

Table S2. Stratified analyses of the associations between dietary vitamin B6 intake with all-cause and cause-specific mortality in men

	Quartile of dietary vitamin B6 intake				P_{trend}	$P_{interaction}$
	Q1	Q2	Q3	Q4		
**All-cause Mortality**						
**Age**						0.138
<65y	Ref	0.95(0.86-1.05)	0.90(0.80-1.01)	0.80(0.68-0.94)	0.004	
≥65y	Ref	0.88(0.81-0.96)	0.83(0.76-0.91)	0.74(0.66-0.82)	<0.001	
**Current smoker**						0.252
Yes	Ref	0.91(0.81-1.02)	0.86(0.75-0.99)	0.78(0.65-0.94)	0.003	
No	Ref	0.91(0.85-0.98)	0.85(0.78-0.93)	0.79(0.71-0.88)	<0.001	
**Current drinker**						0.557
Yes	Ref	0.93(0.86-1.01)	0.89(0.82-0.98)	0.77(0.69-0.87)	<0.001	
No	Ref	0.95(0.86-1.06)	0.84(0.74-0.95)	0.86(0.74-1.01)	0.007	
**BMI**						0.077
<30 kg/m²	Ref	0.93(0.87-1.00)	0.85(0.78-0.92)	0.80(0.72-0.89)	<0.001	
≥30 kg/m²	Ref	0.97(0.86-1.10)	0.93(0.81-1.08)	0.83(0.69-1.00)	0.062	
**Cardiovascular Mortality**						
**Age**						0.014
<65y	Ref	0.92(0.73-1.16)	0.76(0.57-1.03)	0.93(0.64-1.35)	0.194	
≥65y	Ref	0.81(0.69-0.95)	0.74(0.62-0.89)	0.59(0.47-0.74)	<0.001	
**Current smoker**						0.389
Yes	Ref	0.73(0.57-0.94)	0.63(0.46-0.86)	0.59(0.39-0.89)	<0.001	
No	Ref	0.85(0.73-0.98)	0.76(0.64-0.91)	0.69(0.55-0.87)	<0.001	
**Current drinker**						0.450
Yes	Ref	0.92(0.78-1.08)	0.75(0.62-0.92)	0.67(0.52-0.86)	<0.001	
No	Ref	0.86(0.68-1.08)	0.86(0.66-1.14)	0.85(0.59-1.23)	0.289	
**BMI**						0.226
<30 kg/m²	Ref	0.89(0.77-1.03)	0.74(0.62-0.89)	0.71(0.56-0.90)	<0.001	
≥30 kg/m²	Ref	0.78(0.60-1.01)	0.79(0.58-1.07)	0.66(0.44-0.98)	0.020	
**Cancer Mortality**						
**Age**						0.580
<65y	Ref	0.88(0.72-1.07)	1.13(0.90-1.41)	0.76(0.54-1.07)	0.579	
≥65y	Ref	0.79(0.66-0.95)	0.84(0.69-1.01)	0.71(0.56-0.90)	0.005	
**Current smoker**						0.770
Yes	Ref	0.94(0.72-1.22)	0.94(0.69-1.28)	0.68(0.44-1.06)	0.152	
No	Ref	0.79(0.67-0.92)	0.94(0.80-1.11)	0.77(0.62-0.96)	0.049	
**Current drinker**						0.294
Yes	Ref	0.87(0.74-1.02)	1.01(0.84-1.20)	0.67(0.52-0.86)	0.028	
No	Ref	0.84(0.66-1.06)	0.80(0.62-1.05)	0.82(0.59-1.13)	0.114	
**BMI**						0.890
<30 kg/m²	Ref	0.81(0.7-0.95)	0.97(0.83-1.15)	0.7(0.55-0.88)	0.022	
≥30 kg/m²	Ref	1.01(0.78-1.31)	0.9(0.65-1.24)	0.99(0.68-1.44)	0.722	

Adjusted for age, race/ethnicity, BMI, family income-poverty ratio, smoking status (not for smoke stratified analysis), drinking (not for drinking stratified analysis), leisure-time physical activity, total energy intake, diabetes, hypertension, and cardiovascular disease.

Table S3. Stratified analyses of the associations between dietary vitamin B12 intake with all-cause and cause-specific mortality in men

	Quartile of dietary vitamin B12 intake				P_{trend}	$P_{interaction}$
	Q1	Q2	Q3	Q4		
**All-cause Mortality**						
**Age**						0.436
<65y	Ref	1.00(0.90-1.10)	0.99(0.87-1.12)	1.08(0.92-1.25)	0.564	
≥65y	Ref	1.05(0.96-1.14)	0.99(0.90-1.09)	1.02(0.91-1.14)	0.962	
**Current smoker**						0.911
Yes	Ref	1.04(0.91-1.17)	1.09(0.94-1.27)	1.18(0.99-1.41)	0.063	
No	Ref	1.00(0.93-1.08)	0.99(0.91-1.09)	0.96(0.87-1.07)	0.570	
**Current drinker**						0.324
Yes	Ref	1.01(0.93-1.09)	1.01(0.92-1.11)	1.03(0.92-1.15)	0.696	
No	Ref	1.00(0.89-1.12)	1.03(0.90-1.17)	1.05(0.90-1.22)	0.532	
**BMI**						0.963
<24 kg/m^2	Ref	1.00(0.93-1.08)	1.01(0.92-1.10)	1.04(0.93-1.15)	0.580	
≥24 24 kg/m^2	Ref	0.97(0.86-1.11)	1.00(0.86-1.16)	0.98(0.81-1.17)	0.868	
**Cardiovascular Mortality**						
**Age**						0.098
<65y	Ref	1.02(0.80-1.30)	1.00(0.74-1.34)	1.28(0.90-1.83)	0.329	
≥65y	Ref	0.90(0.76-1.06)	0.79(0.65-0.96)	0.83(0.66-1.04)	0.033	
**Current smoker**						0.700
Yes	Ref	1.01(0.77-1.32)	1.06(0.77-1.47)	1.37(0.95-1.99)	0.161	
No	Ref	0.91(0.77-1.07)	0.91(0.75-1.09)	0.80(0.63-1.01)	0.071	
**Current drinker**						0.847
Yes	Ref	0.94(0.79-1.12)	0.95(0.78-1.17)	0.93(0.72-1.19)	0.554	
No	Ref	0.92(0.73-1.15)	0.80(0.61-1.05)	0.98(0.71-1.35)	0.465	
**BMI**						0.401
<30 kg/m^2	Ref	0.99(0.85-1.16)	0.96(0.79-1.16)	0.97(0.76-1.22)	0.683	
≥30 kg/m^2	Ref	0.82(0.63-1.07)	0.77(0.55-1.07)	0.90(0.62-1.32)	0.303	
**Cancer Mortality**						
**Age**						0.706
<65y	Ref	0.87(0.70-1.07)	1.05(0.83-1.32)	0.93(0.68-1.27)	0.257	
≥65y	Ref	1.13(0.94-1.36)	1.01(0.81-1.25)	1.17(0.92-1.49)	0.365	
**Current smoker**						0.786
Yes	Ref	0.99(0.75-1.33)	1.11(0.80-1.55)	1.01(0.67-1.53)	0.749	
No	Ref	0.96(0.82-1.13)	1.05(0.88-1.26)	0.98(0.79-1.22)	0.862	
**Current drinker**						0.970
Yes	Ref	1.00(0.85-1.19)	1.09(0.90-1.31)	1.01(0.80-1.28)	0.664	
No	Ref	1.05(0.82-1.34)	0.99(0.75-1.31)	0.97(0.70-1.35)	0.821	
**BMI**						0.162
<30 kg/m^2	Ref	0.97(0.82-1.13)	1.04(0.87-1.24)	0.90(0.72-1.12)	0.615	
≥30 kg/m^2	Ref	0.83(0.62-1.12)	1.22(0.89-1.66)	1.09(0.75-1.59)	0.317	

Adjusted for age, race/ethnicity, BMI, family income-poverty ratio, smoking status (not for smoke stratified analysis), drinking (not for drinking stratified analysis), leisure-time physical activity, total energy intake, diabetes, hypertension, and cardiovascular disease.

Table S4. Stratified analyses of the associations between dietary folate intake with all-cause and cause-specific mortality in women

	Quartile of dietary folate intake				P_{trend}	$P_{interaction}$
	Q1	Q2	Q3	Q4		
**All-cause Mortality**						
**Age**						0.697
<65y	Ref	0.92(0.82-1.02)	0.89(0.77-1.02)	0.91(0.76-1.08)	0.089	
≥65y	Ref	0.91(0.83-0.99)	0.86(0.78-0.94)	0.84(0.75-0.94)	<0.001	
**Current smoker**						0.277
Yes	Ref	0.94(0.86-1.03)	0.95(0.85-1.05)	0.86(0.75-0.98)	0.034	
No	Ref	0.88(0.79-0.98)	0.80(0.71-0.91)	0.76(0.65-0.89)	<0.001	
**Current drinker**						0.065
Yes	Ref	0.91(0.83-0.99)	0.87(0.78-0.97)	0.80(0.69-0.91)	<0.001	
No	Ref	0.91(0.82-1.01)	0.84(0.74-0.96)	0.90(0.77-1.05)	0.033	
**BMI**						0.765
<30 kg/m^2	Ref	0.91(0.84-0.99)	0.87(0.79-0.96)	0.89(0.79-1.00)	0.006	
≥30 kg/m^2	Ref	0.93(0.83-1.05)	0.89(0.77-1.02)	0.81(0.68-0.96)	0.009	
**Cardiovascular Mortality**						
**Age**						0.650
<65y	Ref	0.95(0.72-1.27)	0.52(0.32-0.84)	0.67(0.39-1.12)	0.015	
≥65y	Ref	0.79(0.66-0.95)	0.68(0.55-0.84)	0.57(0.44-0.75)	<0.001	
**Current smoker**						0.745
Yes	Ref	0.77(0.64-0.94)	0.69(0.54-0.88)	0.54(0.39-0.75)	<0.001	
No	Ref	0.86(0.68-1.10)	0.67(0.49-0.90)	0.53(0.36-0.80)	<0.001	
**Current drinker**						0.642
Yes	Ref	0.84(0.69-1.03)	0.64(0.49-0.83)	0.45(0.31-0.66)	<0.001	
No	Ref	0.85(0.67-1.07)	0.81(0.6-1.10)	0.77(0.51-1.16)	0.120	
**BMI**						0.287
<30 kg/m^2	Ref	0.87(0.73-1.05)	0.71(0.56-0.89)	0.58(0.43-0.79)	<0.001	
≥30 kg/m^2	Ref	0.73(0.57-0.94)	0.65(0.47-0.91)	0.48(0.31-0.76)	<0.001	
**Cancer Mortality**						
**Age**						0.649
<65y	Ref	0.86(0.70-1.05)	1.00(0.78-1.28)	0.79(0.57-1.10)	0.270	
≥65y	Ref	1.03(0.82-1.30)	1.05(0.81-1.35)	0.87(0.64-1.17)	0.532	
**Current smoker**						0.391
Yes	Ref	0.97(0.78-1.21)	1.29(1.01-1.64)	0.92(0.67-1.27)	0.514	
No	Ref	0.83(0.68-1.03)	0.83(0.65-1.06)	0.63(0.46-0.86)	0.004	
**Current drinker**						0.355
Yes	Ref	0.91(0.76-1.10)	0.96(0.77-1.19)	0.77(0.58-1.02)	0.132	
No	Ref	0.98(0.75-1.28)	1.13(0.83-1.53)	0.92(0.62-1.36)	0.976	
**BMI**						0.961
<30 kg/m^2	Ref	0.88(0.72-1.06)	1.05(0.85-1.3)	0.93(0.71-1.23)	0.905	
≥30 kg/m^2	Ref	1.01(0.79-1.28)	1.00(0.75-1.34)	0.62(0.41-0.92)	0.080	

Adjusted for age, race/ethnicity, BMI, family income-poverty ratio, smoking status (not for smoke stratified analysis), drinking (not for drinking stratified analysis), leisure-time physical activity, total energy intake, diabetes, hypertension, and cardiovascular disease.

Table S5. Stratified analyses of the associations between dietary vitamin B6 intake with all-cause and cause-specific mortality in women

	Quartile of dietary vitamin B6 intake				P_{trend}	$P_{interaction}$
	Q1	Q2	Q3	Q4		
**All-cause Mortality**						
**Age**						0.697
<65y	Ref	1.02(0.91-1.13)	0.83(0.73-0.96)	0.87(0.73-1.04)	0.016	
≥65y	Ref	0.90(0.82-0.98)	0.92(0.84-1.01)	0.87(0.77-0.97)	0.013	
**Current smoker**						0.277
Yes	Ref	0.91(0.84-1.00)	0.93(0.84-1.03)	0.92(0.81-1.04)	0.096	
No	Ref	0.98(0.88-1.08)	0.87(0.77-0.98)	0.77(0.66-0.9)	<0.001	
**Current drinker**						0.065
Yes	Ref	0.97(0.89-1.05)	0.83(0.75-0.92)	0.82(0.72-0.94)	<0.001	
No	Ref	0.89(0.80-0.99)	0.98(0.86-1.11)	0.97(0.83-1.15)	0.776	
**BMI**						0.765
<30 kg/m^2	Ref	0.95(0.87-1.03)	0.94(0.86-1.04)	0.92(0.82-1.04)	0.134	
≥30 kg/m^2	Ref	0.94(0.84-1.05)	0.83(0.73-0.95)	0.80(0.67-0.96)	0.002	
**Cardiovascular Mortality**						
**Age**						0.650
<65y	Ref	0.91(0.69-1.21)	0.49(0.32-0.75)	0.48(0.26-0.89)	<0.001	
≥65y	Ref	0.90(0.75-1.07)	0.91(0.74-1.11)	0.58(0.44-0.76)	0.001	
**Current smoker**						0.745
Yes	Ref	0.93(0.77-1.13)	0.82(0.65-1.03)	0.68(0.50-0.92)	0.008	
No	Ref	0.95(0.75-1.21)	0.77(0.58-1.02)	0.40(0.26-0.63)	<0.001	
**Current drinker**						0.642
Yes	Ref	0.89(0.73-1.08)	0.64(0.49-0.82)	0.53(0.37-0.76)	<0.001	
No	Ref	0.93(0.74-1.18)	1.09(0.82-1.46)	0.80(0.54-1.20)	0.666	
**BMI**						0.287
<30 kg/m^2	Ref	0.95(0.79-1.15)	0.76(0.61-0.96)	0.64(0.47-0.85)	0.001	
≥30 kg/m^2	Ref	0.80(0.62-1.02)	0.77(0.57-1.03)	0.46(0.28-0.75)	0.001	
**Cancer Mortality**						
**Age**						0.649
<65y	Ref	0.99(0.81-1.20)	0.85(0.67-1.09)	0.84(0.61-1.15)	0.151	
≥65y	Ref	0.98(0.78-1.24)	0.90(0.70-1.16)	0.87(0.65-1.17)	0.281	
**Current smoker**						0.391
Yes	Ref	1.11(0.90-1.37)	0.98(0.76-1.26)	1.07(0.79-1.45)	0.842	
No	Ref	0.92(0.75-1.13)	0.80(0.63-1.02)	0.65(0.47-0.88)	0.003	
**Current drinker**						0.355
Yes	Ref	1.00(0.84-1.20)	0.82(0.66-1.02)	0.82(0.62-1.08)	0.050	
No	Ref	0.90(0.68-1.18)	1.05(0.78-1.42)	0.98(0.67-1.44)	0.887	
**BMI**						0.961
<30 kg/m^2	Ref	1.01(0.83-1.21)	0.94(0.75-1.16)	0.97(0.74-1.26)	0.638	
≥30 kg/m^2	Ref	1.05(0.83-1.34)	0.89(0.67-1.18)	0.78(0.53-1.13)	0.166	

Adjusted for age, race/ethnicity, BMI, family income-poverty ratio, smoking status (not for smoke stratified analysis), drinking (not for drinking stratified analysis), leisure-time physical activity, total energy intake, diabetes, hypertension, and cardiovascular disease.

Table S6. Stratified analyses of the associations between dietary vitamin B12 intake with all-cause and cause-specific mortality in women

	Quartile of dietary vitamin B12 intake				P_{trend}	$P_{interaction}$
	Q1	Q2	Q3	Q4		
**All-cause Mortality**						
**Age**						0.776
<65y	Ref	1.04(0.93-1.17)	1.09(0.95-1.25)	1.01(0.85-1.2)	0.541	
≥65y	Ref	1.07(0.98-1.17)	1.01(0.91-1.12)	1.06(0.94-1.2)	0.551	
**Current smoker**						0.485
Yes	Ref	1.04(0.95-1.14)	0.99(0.89-1.10)	1.14(1.00-1.30)	0.198	
No	Ref	1.04(0.93-1.15)	1.08(0.95-1.22)	0.98(0.84-1.14)	0.813	
**Current drinker**						0.101
Yes	Ref	1.05(0.96-1.15)	1.07(0.96-1.19)	1.06(0.92-1.21)	0.259	
No	Ref	1.04(0.93-1.16)	0.96(0.85-1.09)	1.05(0.91-1.22)	0.871	
**BMI**						0.054
<30 kg/m²	Ref	1.05(0.97-1.15)	1.07(0.96-1.18)	1.08(0.95-1.22)	0.171	
≥30 kg/m²	Ref	1.02(0.90-1.15)	0.93(0.81-1.07)	1.02(0.85-1.21)	0.727	
**Cardiovascular Mortality**						
**Age**						0.867
<65y	Ref	0.99(0.73-1.35)	0.97(0.67-1.42)	0.67(0.39-1.15)	0.276	
≥65y	Ref	1.23(1.03-1.48)	1.02(0.82-1.27)	1.19(0.90-1.55)	0.392	
**Current smoker**						0.399
Yes	Ref	1.10(0.90-1.34)	0.81(0.63-1.04)	1.13(0.84-1.53)	0.791	
No	Ref	1.13(0.87-1.46)	1.31(0.98-1.77)	1.03(0.70-1.52)	0.344	
**Current drinker**						0.101
Yes	Ref	1.02(0.82-1.26)	1.07(0.83-1.37)	0.84(0.58-1.2)	0.713	
No	Ref	1.26(1.00-1.59)	0.92(0.69-1.22)	1.24(0.89-1.72)	0.577	
**BMI**						0.008
<30 kg/m²	Ref	1.21(0.99-1.47)	1.18(0.93-1.50)	1.22(0.91-1.63)	0.119	
≥30 kg/m²	Ref	0.95(0.73-1.24)	0.64(0.46-0.90)	0.85(0.56-1.29)	0.065	
**Cancer Mortality**						
**Age**						0.547
<65y	Ref	1.09(0.88-1.34)	1.23(0.96-1.56)	1.22(0.9-1.65)	0.099	
≥65y	Ref	0.93(0.73-1.17)	0.85(0.65-1.1)	0.75(0.54-1.03)	0.066	
**Current smoker**						0.109
Yes	Ref	1.29(1.02-1.62)	1.32(1.02-1.71)	1.32(0.96-1.81)	0.045	
No	Ref	0.82(0.66-1.02)	0.84(0.66-1.09)	0.81(0.60-1.10)	0.134	
**Current drinker**						0.560
Yes	Ref	1.04(0.86-1.26)	1.06(0.84-1.32)	1.12(0.85-1.48)	0.418	
No	Ref	0.90(0.68-1.18)	0.89(0.66-1.21)	0.84(0.58-1.21)	0.326	
**BMI**						0.189
<30 kg/m²	Ref	0.96(0.79-1.17)	0.97(0.77-1.22)	1.03(0.79-1.35)	0.932	
≥30 kg/m²	Ref	1.09(0.84-1.41)	1.14(0.85-1.53)	0.95(0.64-1.40)	0.853	

Adjusted for age, race/ethnicity, BMI, family income-poverty ratio, smoking status (not for smoke stratified analysis), drinking (not for drinking stratified analysis), leisure-time physical activity, total energy intake, diabetes, hypertension, and cardiovascular disease.

Table S7 Hazard Ratio (HRs) and 95% CIs for all-cause and cause-specific mortality according to quartiles of dietary folate, vitamin B6, and B12 intake after excluding participants with cardiovascular disease at baseline

	Quartile of nutrient intake				P_{trend}
	**Q1**	**Q2**	**Q3**	**Q4**	
**Men**					
**Folate**					
All-cause mortality	Ref	0.91(0.85-0.97)	0.89(0.82-0.97)	0.80(0.72-0.89)	<0.001
Cardiovascular mortality	Ref	0.86(0.74-1.01)	0.79(0.65-0.95)	0.69(0.54-0.89)	0.001
Cancer mortality	Ref	0.87(0.76-1.00)	0.88(0.75-1.04)	0.74(0.59-0.92)	0.007
**Vitamin B6**					
All-cause mortality	Ref	0.94(0.88-1.01)	0.87(0.81-0.95)	0.80(0.72-0.89)	<0.001
Cardiovascular mortality	Ref	0.87(0.74-1.01)	0.83(0.69-1.00)	0.75(0.58-0.96)	0.007
Cancer mortality	Ref	0.85(0.73-0.98)	0.96(0.82-1.13)	0.77(0.62-0.96)	0.057
**Vitamin B12**					
All-cause mortality	Ref	0.99(0.93-1.07)	0.99(0.91-1.07)	0.99(0.89-1.09)	0.747
Cardiovascular mortality	Ref	0.84(0.71-0.99)	0.88(0.73-1.07)	0.88(0.69-1.11)	0.187
Cancer mortality	Ref	0.98(0.85-1.14)	1.11(0.94-1.31)	0.98(0.80-1.21)	0.675
**Women**					
**Folate**					
All-cause mortality	Ref	0.90(0.83-0.96)	0.87(0.80-0.95)	0.84(0.75-0.93)	<0.001
Cardiovascular mortality	Ref	0.78(0.66-0.92)	0.71(0.57-0.87)	0.49(0.36-0.66)	<0.001
Cancer mortality	Ref	0.93(0.80-1.09)	1.04(0.86-1.24)	0.82(0.65-1.04)	0.305
**Vitamin B6**					
All-cause mortality	Ref	0.94(0.88-1.01)	0.90(0.82-0.97)	0.85(0.77-0.95)	0.001
Cardiovascular mortality	Ref	0.88(0.75-1.04)	0.76(0.62-0.93)	0.53(0.39-0.71)	<0.001
Cancer mortality	Ref	1.02(0.88-1.19)	0.89(0.74-1.07)	0.92(0.73-1.15)	0.230
**Vitamin B12**					
All-cause mortality	Ref	1.03(0.96-1.11)	1.02(0.94-1.12)	1.05(0.95-1.17)	0.376
Cardiovascular mortality	Ref	1.07(0.90-1.28)	1.02(0.82-1.26)	1.01(0.77-1.32)	0.903
Cancer mortality	Ref	1.06(0.90-1.25)	1.03(0.85-1.25)	1.08(0.86-1.36)	0.543

Adjusted for age, race/ethnicity, BMI, family income-poverty ratio, smoking status, drinking, leisure-time physical activity, total energy intake, diabetes, hypertension, and cardiovascular disease.

Table S8 Hazard Ratio (HRs) and 95% CIs for all-cause and cause-specific mortality according to quartiles of dietary folate, vitamin B6, and B12 intake after excluding participants who died within two years of follow-up

	Quartile of nutrient intake				P_{trend}
	**Q1**	**Q2**	**Q3**	**Q4**	
**Men**					
**Folate**					
All-cause mortality	Ref	0.94(0.88-1.00)	0.91(0.84-0.98)	0.85(0.77-0.94)	<0.001
Cardiovascular mortality	Ref	0.85(0.74-0.98)	0.83(0.70-0.98)	0.67(0.54-0.84)	<0.001
Cancer mortality	Ref	0.85(0.74-0.98)	0.84(0.71-0.99)	0.66(0.53-0.83)	<0.001
**Vitamin B6**					
All-cause mortality	Ref	0.96(0.89-1.02)	0.90(0.84-0.97)	0.87(0.79-0.97)	0.001
Cardiovascular mortality	Ref	0.93(0.80-1.06)	0.85(0.72-1.00)	0.86(0.69-1.06)	0.039
Cancer mortality	Ref	0.81(0.70-0.93)	0.93(0.79-1.09)	0.80(0.64-0.99)	0.045
**Vitamin B12**					
All-cause mortality	Ref	1.01(0.94-1.08)	1.02(0.94-1.11)	1.03(0.93-1.14)	0.508
Cardiovascular mortality	Ref	0.92(0.80-1.07)	0.91(0.76-1.08)	0.92(0.74-1.14)	0.311
Cancer mortality	Ref	0.97(0.84-1.13)	1.08(0.91-1.28)	0.99(0.80-1.22)	0.730
**Women**					
**Folate**					
All-cause mortality	Ref	0.92(0.86-0.99)	0.87(0.8-0.95)	0.86(0.78-0.96)	<0.001
Cardiovascular mortality	Ref	0.84(0.71-0.98)	0.70(0.57-0.85)	0.56(0.43-0.74)	<0.001
Cancer mortality	Ref	0.93(0.79-1.09)	1.03(0.86-1.23)	0.84(0.66-1.06)	0.357
**Vitamin B6**					
All-cause mortality	Ref	0.96(0.89-1.02)	0.92(0.85-1.00)	0.87(0.79-0.97)	0.004
Cardiovascular mortality	Ref	0.92(0.79-1.08)	0.78(0.64-0.94)	0.53(0.40-0.70)	<0.001
Cancer mortality	Ref	1.02(0.87-1.19)	0.97(0.81-1.16)	0.87(0.69-1.09)	0.297
**Vitamin B12**					
All-cause mortality	Ref	1.04(0.97-1.12)	1.04(0.96-1.13)	1.04(0.93-1.15)	0.383
Cardiovascular mortality	Ref	1.13(0.96-1.33)	0.99(0.81-1.21)	1.03(0.80-1.33)	0.951
Cancer mortality	Ref	1.05(0.89-1.23)	1.10(0.92-1.33)	1.03(0.81-1.30)	0.559

Adjusted for age, race/ethnicity, BMI, family income-poverty ratio, smoking status, drinking, leisure-time physical activity, total energy intake, diabetes, hypertension, and cardiovascular disease.

Table S9 Hazard Ratio (HRs) and 95% CIs for all-cause and cause-specific mortality according to quartiles of dietary folate, vitamin B6, and B12 intake further adjusting folate supplement.

	Quartile of nutrient intake				P_{trend}
	**Q1**	**Q2**	**Q3**	**Q4**	
**Men**					
**Folate**					
All-cause mortality	Ref	0.93(0.87-0.99)	0.89(0.83-0.96)	0.81(0.74-0.90)	<0.001
Cardiovascular mortality	Ref	0.89(0.78-1.02)	0.87(0.74-1.03)	0.76 (0.61-0.95)	0.011
Cancer mortality	Ref	0.87(0.76-0.99)	0.89(0.76-1.04)	0.68(0.55-0.83)	0.001
**Vitamin B6**					
All-cause mortality	Ref	0.95(0.89-1.01)	0.88(0.82-0.95)	0.82(0.74-0.91)	<0.001
Cardiovascular mortality	Ref	0.91(0.80-1.03)	0.83(0.71-0.970)	0.85(0.69-1.05)	0.021
Cancer mortality	Ref	0.85(0.74-0.97)	0.96(0.83-1.11)	0.74(0.60-0.91)	0.023
**Vitamin B12**					
All-cause mortality	Ref	1.00(0.94-1.07)	1.02(0.94-1.10)	1.02(0.93-1.11)	0.633
Cardiovascular mortality	Ref	0.91(0.79-1.04)	0.94(0.80-1.10)	0.95(0.78-1.16)	0.500
Cancer mortality	Ref	0.97(0.84-1.11)	1.09(0.93-1.27)	0.96(0.79-1.16)	0.867
**Women**					
**Folate**					
All-cause mortality	Ref	0.92(0.86-0.99)	0.91(0.83-0.99)	0.89(0.80-0.99)	0.011
Cardiovascular mortality	Ref	0.9(0.77-1.04)	0.87(0.72-1.06)	0.71(0.54-0.93)	0.014
Cancer mortality	Ref	0.94(0.81-1.1)	1.09(0.9-1.31)	0.86(0.67-1.09)	0.583
**Vitamin B6**					
All-cause mortality	Ref	0.93(0.87-1)	0.91(0.84-0.99)	0.9(0.82-1)	0.012
Cardiovascular mortality	Ref	0.93(0.8-1.08)	0.85(0.71-1.02)	0.69(0.53-0.9)	0.005
Cancer mortality	Ref	1.01(0.87-1.17)	0.92(0.77-1.09)	0.91(0.72-1.13)	0.266
**Vitamin B12**					
All-cause mortality	Ref	1.03(0.96-1.11)	1.04(0.96-1.13)	1.07(0.97-1.19)	0.155
Cardiovascular mortality	Ref	1.1(0.94-1.28)	1.04(0.86-1.26)	1.09(0.86-1.39)	0.486
Cancer mortality	Ref	1.03(0.88-1.2)	1.06(0.88-1.27)	1.05(0.84-1.31)	0.560

Adjusted for age, race/ethnicity, BMI, family income-poverty ratio, smoking status, drinking, leisure-time physical activity, total energy intake, diabetes, hypertension, cardiovascular disease, and folate supplement.
